# Protection of Vaccination Wounds

**Published:** 1920-07-17

**Authors:** 


### Protection of Vaccination Wounds.

We see described in a recent number of the American *Western Medical Times* a simple and practical form of dressing for vaccination wounds, applicable at any stage of their progress. Apart from the actual utility of the dressing, there is value in the fact that the chief components are flat pieces of discarded wooden tongue-depressers. A broad V is cut in the edges of the wood and two Vs approximated so as to form a diamond-shaped opening in which the vaccination wound may lie and be extremely well sheltered. A couple of strips of adhesive plaster will retain the apparatus firmly in its place.

A Novel Form of Dressing.

Also, on the typical case, the light wooden pieces tend to indent and bury themselves in the slightly œdematous skin, thus securing the position yet more firmly. It is explained that when these pieces are properly placed the bandage may be changed frequently or seldom, neither contact, adhesion nor friction need be considered. Antiseptic dressings may be applied above the shield after the typical effect of the virus has been observed and allowed to proceed for a few days. In a sketch of other treatment applicable in severe instances of extensive vaccination wound these are mentioned: Lead-wash, a variety of local measures, and the use of polyvalent or autogenous vaccines, but the point is made that all this may be carried out without disturbing or moving the little wooden protectors, and that thereby the comfort of the patient is considerably increased.

